# Fecal and Duodenal Microbiota in Pediatric Celiac Disease

**DOI:** 10.3389/fped.2021.652208

**Published:** 2021-04-22

**Authors:** Diyora Abdukhakimova, Kuanysh Dossybayeva, Dimitri Poddighe

**Affiliations:** ^1^Department of Medicine, Nazarbayev University School of Medicine (NUSOM), Nur-Sultan, Kazakhstan; ^2^Department of Pediatrics, National Research Center for Maternal and Child Health, University Medical Center, Nur-Sultan, Kazakhstan

**Keywords:** celiac disease, children, gut microbiota, microbiome, HLA-DQ, *Bifidobacterium* spp

## Abstract

**Background and Objective:** The gut microbiota plays a role in regulating the host immunity. Therefore, alterations in gut microbiota (or dysbiosis) have been investigated in several gastrointestinal diseases, including Celiac Disease (CD). The aim of this study is to summarize the main characteristics of the gut microbiota in pediatric CD.

**Methods:** We performed a systematic review to retrieve the available studies investigating the gut microbiota in pediatric CD patients and controls. In detail, after the screening of >2,200 titles from the medical literature, 397 articles were assessed for eligibility based on the abstracts: of those, 114 full-text original articles were considered as eligible according to the aim of this systematic review.

**Results:** The final search output consisted of 18 articles describing the gut microbiota of CD children and including one or more control groups. Eleven pediatric studies provided information on the duodenal microbiota and as many investigated the fecal microbiota; three articles analyzed the microbiota on both fecal and duodenal samples from the same cohorts of patients.

**Conclusion:** Due to the heterogeneity of the experimental procedures and study design, it is not possible to evidence any specific celiac signature in the fecal and/or duodenal microbiota of CD children. However, some specific components of the fecal microbiota and, in detail, *Bifidobacterium* spp. (e.g., *Bifidobacterium longum*) may deserve additional research efforts, in order to understand their potential value as both probiotic therapy and diagnostic/prognostic biomarker.

## Introduction

The microbial communities naturally colonizing the gastrointestinal (GI) tract, namely, gut microbiota, are an essential and physiological component of a healthy human body. In fact, the “whole” deriving from this symbiotic interaction between microbiota and host, has been defined as a “superorganism,” where the former contributes to several metabolic, physiologic, inflammatory, and immunologic functions for the latter (1, 2).

In detail, the microbiota plays a role in regulating the host immunity by influencing the development and homeostasis of the gut epithelial layer and mucosal-associated lymphoid tissue. Therefore, alterations in gut microbiota (or dysbiosis) have been explored and investigated in several GI disorders, including Celiac Disease (CD).

Indeed, CD is a systemic immune-mediated condition, characterized by a variable pattern of GI and extra-GI clinical manifestations, but clearly defined by the presence of gluten-dependent atrophic (small bowel) enteropathy associated with a consistent serological panel (positivity for anti-tissue transglutaminase antibody and/or anti-endomysium antibody) (3, 4). Importantly, gluten can trigger CD in a minority of patients who are carriers of specific HLA-DQ genotypes (HLA-DQ2 and HLA-DQ8): indeed, both these environmental and genetic factors respectively represent necessary, but not sufficient, conditions to develop CD (5).

Each GI tract is characterized by a peculiar microbiota, in terms of qualitative and quantitative composition. In general, the bacterial density progressively increases along the GI tract, ranging from 10^3^ units per gram of luminal content in the duodenum up to values of 10^12^ order in the colon; concomitantly, the bacterial diversity (in terms of number of different bacterial species) gradually increases from the proximal to the distal GI tracts as well (2, 6).

The knowledge on gut microbiota has greatly improved in the last few years, since culture-independent approaches became available, such as the analysis of bacterial 16S ribosomal RNA (rRNA) gene. Among bacteria (indeed, also yeast and viruses are part of gut microbiota), the dominant phyla are Firmicutes and Bacteroidetes: the former phylum mainly includes *Clostridium* spp., *Bacillus* spp., *Lactobacillus* spp., *Ruminococcus* spp., and *Enterococcus* spp.; Bacteroidetes phylum is predominantly composed of *Bacteroides* spp. and *Prevotella* spp. The remaining phyla (Actinobacteria, Fusobacteria, Proteobacteria, and Verrucomicrobia) represent around 10% of the bacterial gut population; among them, the most studied genus is *Bifidobacterium* spp., which belongs to Actinobacteria phylum (7–9).

The inter-individual variability of gut microbiota is high: indeed, its composition is influenced by a multitude of factors, such as genetics, age, diet, hygiene level, and medication exposure (first of all, antibiotics). As mentioned above, deviations in the composition of gut microbiota may also be related to specific pathological conditions, but it is still unclear if these are cause or effect of the comorbid disease, due to the limitations of the available studies so far (10, 11).

In this systematic review, we summarized the main characteristics of the gut microbiota in pediatric CD patients.

## Materials and Methods

### Protocol

The PRISMA guidelines were used for this systematic review (12). This systematic review includes original articles (such as experimental studies, randomized control trials, case–control studies, cohort studies, and observational studies), providing information on gut microbiota in CD children and age-matched controls. Indeed, the primary endpoint was the description of gut microbiota in pediatric patients affected with CD. This systematic search did not include review and abstract papers.

### Search Strategy

The literature search strategy of this review consisted of two stages: (i) an extensive search in five databases, namely, Scopus, Cochrane Central Register of Controlled Trials, PubMed, Ovid, and Web of Science, by using relevant keywords, as described in Table 1; (ii) a search of reference lists from the collected and related articles identified at the previous stage.

**Table 1:** Search strategy of the systematic review.

**Database**	**Search strategy**
SCOPUS	(TITLE-ABS-KEY (“celiac disease” OR “coeliac”) AND TITLE-ABS-KEY (“gut microbiota” OR “bacteria” OR “microbes” OR “microbiome” OR “microbiota dysbiosis” OR “metagenome” OR “metabolomics” OR “fecal microbiota” OR “intestinal microbiota” OR “duodenal microbiota”)
PUBMED	(“celiac disease” [mh] OR “coeliac” [mh]) AND (“gut microbiota” OR “bacteria” OR “microbes” OR “microbiome” OR “microbiota dysbiosis” OR “metagenome” OR “metabolomic” OR “fecal microbiota” OR “intestinal microbiota” OR “duodenal microbiota”), showing the search details: “celiac disease” [mh] AND (“gut microbiota” [All Fields] OR “bacteria” [All Fields] OR “microbes” [All Fields] OR “microbiome” [All Fields] OR “microbiota dysbiosis” [All Fields] OR “metagenome” [All Fields] OR “metabolomics” [All Fields] OR “fecal microbiota” [All Fields] OR “intestinal microbiota” [All Fields] OR “duodenal microbiota” [All Fields])
COCHRANE	*“celiac disease” OR “coeliac” in Title Abstract Keyword AND “gut microbiota” OR “bacteria” OR “microbes” OR “microbiome” OR “microbiota dysbiosis” OR “metagenome” OR “metabolomics” OR “fecal microbiota” OR “intestinal microbiota” OR “duodenal microbiota” in Abstract - (Word variations have been searched)
OVID	(celiac disease OR coeliac) AND (gut microbiota OR bacteria OR microbes OR microbiome OR microbiota dysbiosis OR metagenome OR metabolomic OR fecal microbiota OR intestinal microbiota OR duodenal microbiota). The exact search details were: (celiac disease or coeliac) and (gut microbiota or bacteria or microbes or microbiome or microbiota dysbiosis or metagenome or metabolomic or fecal microbiota or intestinal microbiota or duodenal microbiota) [mp = title, abstract, original title, name of substance word, subject heading word, floating sub-heading word, keyword heading word, organism supplementary concept word, protocol supplementary concept word, rare disease supplementary concept word, unique identifier, synonyms] {Including Related Terms}.
WEB OF SCIENCE	1- (TS = (celiac disease OR coeliac disease)) AND LANGUAGE: (English) AND DOCUMENT TYPES: (Article); 2- (ALL = (gut microbiota OR bacteria OR microbes OR microbiome OR microbiota dysbiosis OR metagenome OR metabolomic OR fecal microbiota OR intestinal microbiota OR duodenal microbiota)) AND LANGUAGE: (English) AND DOCUMENT TYPES: (Article); 3- #2 AND #1.

### Data Extraction

After a critical reading of the articles, data extraction was done by one investigator and then checked by a second investigator following these inclusion criteria: any original articles in which gut microbiota composition was studied in CD patients with clear description of methods and outcomes. In detail, the following items were extracted from each study: first author's last name, publication year, country of origin, study population details, age group, sample type, aims of the study, intervention type, number of participants, and analytical methods to study microbiota.

## Results

**Figure 1:**
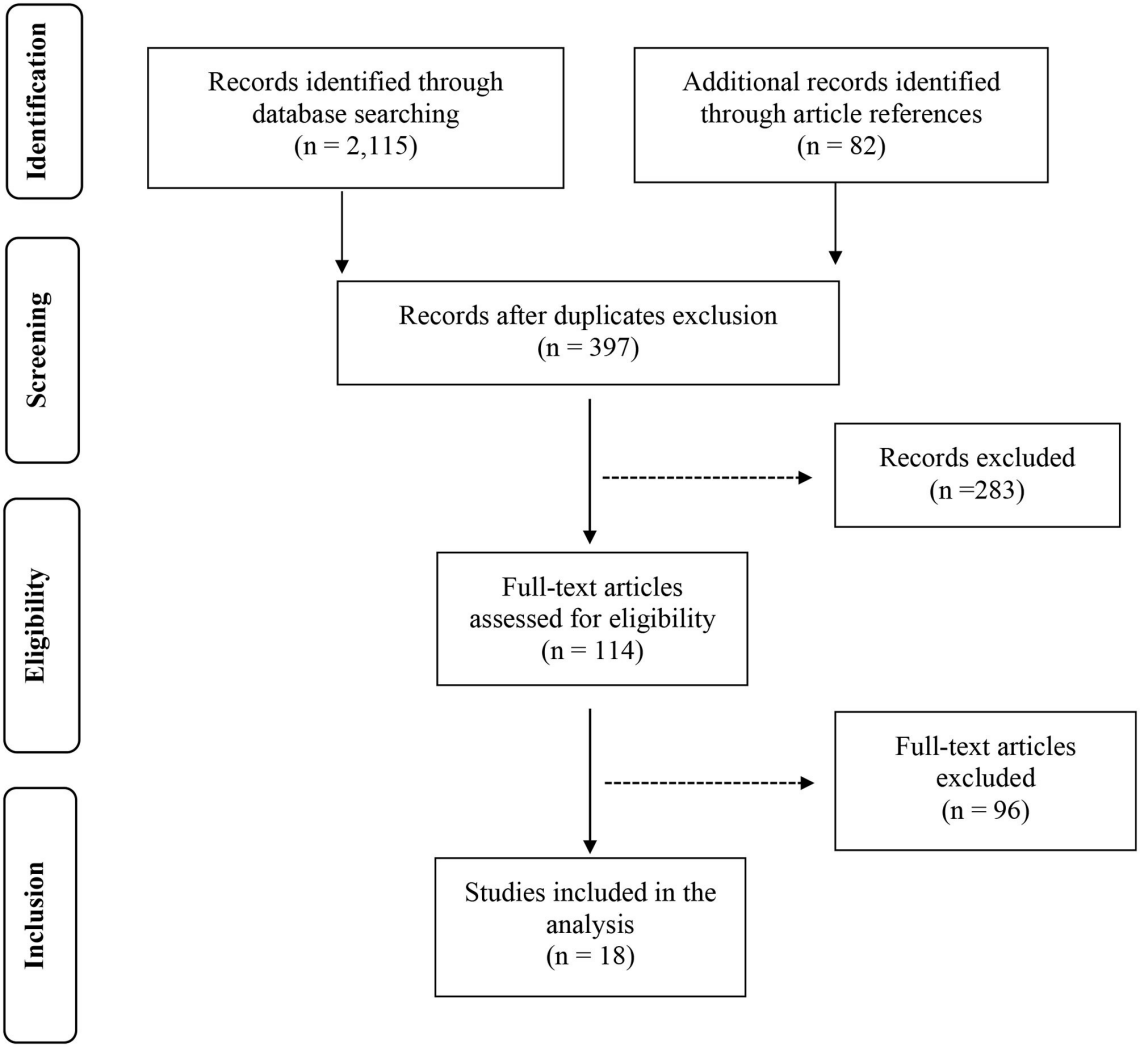
PRISMA flow diagram of the systematic review.

The results of each step of the literature search according to the PRISMA guidelines are schematically summarized in Figure 1. In detail, the literature search resulted in 1,143 papers from Scopus, 44 from the Cochrane Central Register of Controlled Trials, 337 from PubMed, 82 from Ovid, and 509 from Web of Science. Eighty-two additional articles were identified through the references of the previous papers. After the screening by title, the duplicated records were removed, and 397 articles were assessed for eligibility based on the abstracts: of those, 114 full-text original articles were considered as eligible according to the aim of this systematic review. The final search output consisted of 18 articles describing the gut microbiota of CD children and including one or more control groups (13–30). All the articles finally included in this systematic review are listed in Table 2, where the study population, participants' age group, sample type(s), aim of the study, type of intervention, sample size, and detailed laboratory methods are summarized. Importantly, studies focused on HLA-DQ predisposition, which analyzed the gut microbiota in children before becoming celiac (thus, with no microbiome analysis in CD pediatric patients after the diagnosis), were excluded. Moreover, only studies including a control group to be compared to CD children, were included. The specific findings resulting from these selected studies are summarized in Tables 3 and 4, which refer to the gut microbiota studies on duodenal biopsy and stool samples, respectively. In detail, these tables provide the main findings in terms of overall bacterial abundance and/or bacterial diversity and/or specific bacterial composition, according to the variable aims of each study.

**Table 2:** Overview of the articles included in the systematic review.

**#**	**References**	**Country**	**Study** **population**	**Age**	**Sample** **type**	**Aims of** **the study**	**Intervention**	**Number of** **participants**	**Methods to study microbiota**
1	Collado et al. (13)	Spain	1) Untreated CD 2) Controls (age matched non-CD children)	Mean (range): 1) 26.3 (12–48) mo. 2) 23.0 (11–45) mo.	Fecal sample	Comparison of fecal microbiota in CD and control children.	Observational	1) *n* = 26 2) *n* = 23	The fecal bacteria were analyzed using different plate culture media. Cellular morphology, Gram staining, biochemical test and antibiotic susceptibility analysis, were used to identify bacterial colonies. Total counts of bacteria were expressed as log of the number of colonies forming units (CFU) per gram of wet feces.
2	Nadal et al. (14)	Spain	1) Active CD 2) Inactive CD (on GFD for 1-2 years) 3) Controls (without known food intolerance)	Mean (range): 1) 5.1 yrs. (1.6–12.0) 2) 5.6 yrs. (2.0–7.8) 3) 4.1 yrs. (1.9–9.0)	Duodenal samples	Determination of duodenal microbiota composition of active CD children and symptom-free CD patients on GFD and healthy controls.	Observational	1) *n* = 20 2) *n* = 10 3) *n* = 8	Fluorescent *in situ* hybridization (FISH) and flow cytometry with oligonucleotide probes was used to identify bacterial groups.
3	Sanz et al. (15)	Spain	1) Active CD 2) Controls (without known food intolerance)	Mean (range): 1) 28 (15–45) mo. 2) 24 (11–40) mo.	Fecal sample	Identification of difference in *Bifidobacterium* and *Lactobacillus* groups in microbiota of celiac children and controls.	Observational	1) *n* = 10 2) *n* = 10	The microbiome analysis was performed by denaturing gradient gel electrophoresis (DGGE) analyses for *Bifidobacterium* spp. and *Lactobacillus* spp. using universal and specific primers.
4	Collado et al. (16)	Spain	1) Active CD patients 2) Inactive CD (after GFD) 3) Controls (without gluten intolerance)	Mean (SD): 1) 56.4 (38.5) mo. 2) 65.2 (37.7) mo. 3) 45.0 (33.5) mo.	Fecal samples & duodenal samples	Assessment of Bifidobacteria composition of fecal samples from CD patients and controls	Observational	1) *n* = 30 2) *n* = 18 3) *n* = 30	DNA extraction was done by the QIAamp DNA stool Mini kit. Real time PCR was done by the ABI PRISM 7000-PCR sequence detection system. The Ct values obtained from standard curves were used to find the bacterial concentration of samples analyzed.
5	Collado et al. (17)	Spain	1) Untreated CD 2) Treated CD 3) Controls (without gluten intolerance)	Range: 1) 56.4–60.6 mo. 2) 63.5-57.8 mo. 3) 45.0-49.2 mo.	Fecal samples & duodenal samples	Identification of specific gut bacteria in CD at diagnosis and after GFD in children.	Observational	1) *n* = 30 2) *n* = 18 3) *n* = 30	DNA extraction was done using the QIAamp DNA stool Mini kit. Real time PCR was done by the ABI PRISM 7000-PCR sequence detection system. The Ct value comparison was done to find the bacterial concentration for each sample.
6	Di Cagno et al. (18)	Italy	1) Inactive CD (after GFD) 2) Active CD (after and despite GFD 3) Controls (without known food intolerance)	Range: 6–12 yrs.	Fecal sample	Description of the differences in the fecal microbiota between treated and untreated CD children and their controls.	Observational	1) *n* = 7 2) *n* = 7 3) *n* = 7	DNA extraction was done by the FastDNA Pro Soil-Direct kit. PCR was performed by using the eubacterial universal and group-specific 16S rRNA gene primers. The microbiome analysis was done by the denaturing gradient gel electrophoresis (DGGE) analyses.
7	Sánchez et al. (19)	Spain	1) Active CD 2) Treated CD 3) Controls (without gluten intolerance)	Mean (SD): 1) 51.1 (31.8) mo. 2) 54.9 (25.6) mo. 3) 50.1 (31.2) mo.	Duodenal samples	Characterization of bacterial populations associated with duodenal biopsies of children with active and treated CD.	Observational	1) *n* = 20 2) *n* = 12 3) *n* = 8	DNA extraction was done by the QIAamp DNA stool Mini kit. Bacterial populations were analyzed by PCR amplification and DGGE.
8	De Palma et al. (20)	Spain	1) Untreated CD 2) Treated CD 3) Controls (without gluten intolerance)	Mean (range): 1) 5.5 yrs. (2.1–12.0) 2) 5.5 yrs. (1–12.3) 3) 5.3 yrs. (1.8–10.8)	Fecal sample	Evaluation of the relationships between fecal microbiota composition and immunoglobulin-coated bacteria in untreated and treated CD children.	Observational	1) *n* = 24 2) *n* = 18 3) *n* = 20	The oligonucleotide probes were used for the fluorescent *in situ* hybridization (FISH) analysis identifying bacteria colonizing gut. Flow cytometry was used to identify immunoglobulin-coated bacteria, through EPICS^®^ XL-MCL flow cytometer.
9	Schippa et al. (21)	Italy	1) Active CD 2) Remission state CD 3) Controls (undergoing the endoscopy for functional dyspepsia)	Mean (range): 1) 8.3 yrs. (1.2–16.1) 2) 8.3 yrs. (1.2–16.1) 3) 11.7 yrs. (7.8–20.8)	Duodenal samples	Studying the influence of the CD status on the microbial composition.	Observational	1) *n* = 20 2) *n* = 20 3) *n* = 10	DNeasy tissue kit was used for DNA extraction. The 16S rDNA gene-targeted primer PCR was performed before the sequence-specific separation of PCR products using the DCode Universal mutation detection system.
10	Di Cagno et al. (22)	Italy	1) Treated CD 2) Non-CD (without known food intolerance, undergoing the endoscopy for functional dyspepsia)	Range: 6–12 yrs.	Fecal samples & duodenal samples	Evaluating the difference in the composition of microbiota and metabolome between treated CD children and healthy controls.	Observational	1) *n* = 19 2) *n* = 15	The microbiota and some subgroups (e.g., Bifidobacteria and Lactobacilli) were analyzed by PCR (universal primers targeting V6-V8 regions of the 16S rRNA) and denaturing gradient gel electrophoresis (DGGE).
11	Nistal et al. (23)	Spain	Children: 1) Untreated CD 2) Controls (without known food intolerance) Adults: 1) Untreated CD 2) Treated CD 3) Controls (without known food intolerance)	Mean (range) Children: 1) 3.75 yrs. (1–10) 2) 7.2 yrs. (3–12) Adults: 1) 31.4 yrs. (26–38) 2) 18.8 yrs. (16–21) 3) 29.2 yrs. (15–40)	Duodenal samples	Assessment of the bacterial composition in the upper small intestine of adults and children.	Observational	Children: 1) *n* = 8 2) *n* = 5 Adults: 1) *n* = 5 2) *n* = 5 3) *n* = 5	DNA extraction was done by NucleoSpin Tissue XS kit. PCR with 16S rRNA gene primers was performed for sequencing and identification using phylogenetic analysis.
12	Kalliomäki et al. (24)	Finland	1) CD children with active CD 2) CD adults on GFD (>1 yrs.) 3) Control children (without known food intolerance)	Mean (range) 1) 9.5 yrs. (3–14) 2) 46 yrs. (30–60) 3) 8.5 yrs. (4–16)	Duodenal samples	Evaluation of microbiota, its Toll-like receptors, and their regulators in the small intestinal mucosa in CD.	Observational	1) *n* = 10 2) *n* = 6 3) *n* = 9	The analysis of microbiota was done by performing the quantitative PCR through Applied Biosystems 7300 Fast Real-Time PCR System in a 96-well format and using SYBR Green chemistry primers. The DNA was extracted from biopsy samples with Bead Beating and the Qiagen column. The results were analyzed comparing the Ct values of samples with those of the standard curves.
13	Sánchez et al. (25)	Spain	1) Active CD 2) Non-active CD 3) Controls (without known food intolerance)	Mean (SD): 1) 57.4 mo. (37.6) 2) 67.3 mo. (38.4) 3) 54.0 mo. (34.1)	Fecal samples	Determining differences in the *Staphylococcus* spp. and their characteristics between CD patients and healthy controls.	Observational	1) *n* = 20 2) *n* = 20 3) *n* = 20	Staphylococci were isolated from fecal samples and identified by PCR using the primers for *Staphylococcus* isolates and DNA sequencing with an ABI PRISM-3130XL Gene Analyzer.
14	Cheng et al. (26)	Finland	1) Active CD children 2) Controls (children having EGDS) 3) CD adults after GFD	Mean ± SD (range): 1) 9.5 ± 4.1 yrs. (3–14) 2) 8.5 ± 3.8 yrs. (4–16) 3) 46 ± 11.4 yrs. (30–60)	Duodenal samples	Complete duodenal mucosal microbiota characterization and assessment of the differences in the microbiota of CD patients and healthy controls.	Observational	1) *n* = 10 2) *n* = 10 3) *n* = 6	The microbiota was analyzed through bacterial phylogenetic microarray HITChip (Human Intestinal Tract Chip) consisting of over 4,800 oligonucleotide probes. The abundance of Gram-positive, Gram-negative, or flagellated bacterial groups was summarized using HITChip profiles.
15	Sánchez et al. (27)	Spain	1) Active CD 2) Non-active CD 3) Controls (without known food intolerance)	Mean (SD): 1) 5.9 yrs. (3.2) 2) 5.9 yrs. (1.2) 3) 6.9 yrs. (4.2)	Duodenal samples	Studying whether live culture-dependent bacteria related to duodenal mucosa in active and non-active CD patients and controls have different composition.	Observational	1) *n* = 32 2) *n* = 17 3) *n* = 8	Samples were randomly plated on two different media: plate count agar, Wilkins-Chalgren agar, brain heart agar yeast, Casitone, and fatty acid agar. Bacterial isolates underwent bacterial DNA extraction for the 16S rRNA gene PCR amplification.
16	Pisarello et al. (28)	Argentina	1) Controls (no family history of food intolerance) 2) Symptom-free children (previously confirmed CD and GFD)	Mean (range) 1) 6.5 (2–11) yrs. 2) 7.5 (3–14) yrs.	Fecal samples	Identification of most common groups of bacteria in the intestinal microbiota of symptom-free CD children on GFD and healthy controls.	Observational	1) *n* = 15 2) *n* = 15	Samples were diluted and aliquoted in plates for lactobacilli, enterobacteria, and total aerobic bacteria. Gram staining, catalase test, appearance of colony and cell morphology, and fermentation profiles of carbohydrates, were used to identify the lactobacilli.
17	Quagliariello et al. (29)	Slovenia	1) Probiotic group before treatment (B. breve BR03 and B. breve B632) 2) Placebo group before treatment 3) Controls (without known food intolerance)	Range: 1–19 yrs.	Fecal samples	Evaluation of the effects of the administration *Bifidobacterium breve* strains (B632 and BR03) on gut microbiota in CD pediatric patients on a GFD.	Randomized placebo controlled trial	1) *n* = 20 2) *n* = 20 3) *n* = 16	DNA extraction was done by using the QIAamp DNA Stool Mini Kit. qPCR using Fast SYBR^®^ Green Master Mix was done to identify *Bifidobacterium* spp., *Lactobacillus* spp., *Bacteroides fragilis, Clostridium* group and total enterobacteria. Illumina MiSeq Sequencing was used to assess the abundance of specific bacterial groups.
18	Di Biase et al. (30)	Italy	1) CD 2) Controls (non-CD)	Range: 1–18 yrs.	Fecal samples & duodenal samples	Assessment of the microbiota composition in CD children at diagnosis and the relationship between bacterial abundance and symptoms.	Observational	1) *n* = 21 2) *n* = 16	DNA extraction from fecal samples was done by DNeasy Blood and Tissue Mini kit; the same kit was used for the duodenal samples using modified protocol. PCR was done using the T7prom-Bact-27-F and Uni-1492-R primers with 16S rRNA gene primers.

*GFD, gluten free diet; yrs., years; mo., months; EGDS, esophago-gastro-duodenal endoscopy*.

**Table 3:** Main findings from the studies investigating the duodenal microbiota.

**#**	**References**	**Microbiome composition results**
1	Nadal et al. (14)	*Bacteroides–Prevotella* bacteria and *E. coli* were significantly more abundant in biopsy specimens of CD patients with active disease than in controls. These results were not significantly different between controls and symptom free CD patients. *Bacteroides–Prevotella, Streptococcus–Lactococcus*, and *E. coli* were dominant groups in active CD patients, while in non-active CD patients the dominant groups were *Streptococcus–Lactococcus* and *Clostridium histolyticum*; in control children, *Clostridium histolyticum, Bifidobacterium*, and *Faecalibacterium prausnitzii* were the most abundant groups.
2	Collado et al. (16)	*Bifidobacterium longum* was one of the most frequently detected, then it were the following frequently detected species: *Bifidobacterium breve, Bifidobacterium bifidum, Bifidobacterium catenulatum*, and *Bifidobacterium lactis*. In active CD group and control patients' biopsy samples more prevalent species was *Bifidobacterium lactis*. The prevalence of total *Bifidobacterium* was statistically significantly higher in controls than in active CD group.
3	Collado et al. (17)	The highest total bacterial counts (not statistically significant) were in the untreated CD group, followed by treated CD patients and, finally, controls. *Clostridium coccoides* group was more prevalent in controls than in the other two groups (treated, not treated CD). *Lactobacillus* spp. was more prevalent in untreated CD patients and in controls, compared to treated CD group. *Akkermansia muciniphila* was more prevalent in untreated CD patients compared to treated CD. *Bacteroides* and *Clostridium leptum* groups were more prevalent in CD groups compared to controls. *Staphylococcus* and *E. coli* groups were more prevalent in untreated CD than treated CD and controls. *Bifidobacterium* spp. was more prevalent in controls than in untreated CD patients. *Lactobacillus* group levels were significantly lower in treated CD control groups than in untreated CD.
4	Sánchez et al. (19)	*Bacteroides distasonis, Bacteroides fragilis/Bacteroides thetaiotaomicron, Bacteroides uniformis*, and *Bacteroides ovatus* were higher in controls than in CD patients. *Bacteroides vulgatus* was higher in controls than in treated CD patients. *Bacteroides dorei* was more frequent in active CD patients than in treated CD patients and controls. *Bifidobacterium adolescentis* and *Bifidobacterium animalis* were higher in active CD than in treated CD and controls. A higher Lactic Acid Bacteria (LAB) diversity was described in treated CD and control patients than in active CD group. *Weissella* spp. and *Lactobacillus fermentum* were more common in treated CD than in controls and active CD patients.
5	Schippa et al. (21)	Statistically significant differences between CD patients and controls were respectively found in the prevalence of *Bacteroides vulgatus* (85 vs. 20%), and *E. coli* (95 vs. 20%). Additionally, significant differences were found in the prevalence of *Bacteroides vulgatus* (80 vs. 90%) and *Clostridium coccoides* group (50 vs. 90%) between in active and inactive CD patients, respectively. There was no statistically significant difference in the prevalence of *Bifidobacterium* spp. between CD patients and control group, and between active and inactive CD patients. Overall, bacterial population was more diverse in duodenal mucosa of CD group compared to the control group.
6	Di Cagno et al. (22)	The *Lactobacillus plantarum* was present in duodenal biopsies of treated CD patients and healthy controls. *Bifidobacteria* group were not present in duodenal biopsies of these groups.
7	Kalliomäki et al. (24)	There was no statistically significant difference in the bacterial species between the following groups and species: *Bifidobacterium* genus, *Bifidobacterium adolescentis, Bifidobacterium catenulatum* group, *Bifidobacterium longum* subsp *infantis, Bifidobacterium longum, Staphylococcus aureus, Bacteroides*-*Prevotella*-*Porphyromonas* group, *Bacteroides fragilis* group, *Streptococcus* genus, and *Lactobacillus* group. The *Bacteroides fragilis* group, *Bifidobacterium catenulatum* group, *Bifidobacterium longum*, and *Streptococcus* genus were detected in a small number of samples. However, *Lactobacillus* group, *Staphylococcus aureus*, and *B. longum* subsp *infantis* were not detected at all.
8	Nistal et al. (23)	Ninety eight percent of the sequenced bacterial species from the proximal small intestine of adults belonged to the following phyla: *Firmicutes* (38%), *Proteobacteria* (29%), *Bacteroidetes* (17%), *Actinobacteria* (10%), and *Fusobacteria* (4%). Majority (99%) of the sequences among children belonged to *Proteobacteria* (38%), *Firmicutes* (34%), *Bacteroidetes* (13%), *Actinobacteria* (4%), *Deinococcus-Thermus* (2.7%), *Fusobacteria* (2.9%), and unknown phylum sequences (5%). Sequences from genera such as *Streptococcus, Prevotella, Neisseria, Haemophilus, Granulicatella*, and *Acinetobacter* were present in at least 60% of children. *Streptococcus* and *Prevotella* genera sequences were more prevalent among healthy children compared to untreated CD children.
9	Cheng et al. (26)	In both healthy controls and CD groups, major bacterial groups were found: *Sutterella wadsworthensis* et rel., *Streptococcus mitis* et rel., *Aquabacterium, Streptococcus bovis* et rel., *Streptococcus intermedius* et rel., and *Prevotella melaninogenica* et rel. *Sutterella wadsworthensis* et rel. and *Streptococcus mitis* et rel. were the most abundant ones. *Haemophilus* spp. and *Serratia* spp. were higher in CD group than in healthy controls.
10	Sánchez et al. (27)	Members of phylum *Proteobacteria* were higher in active CD patients than in controls and non-active CD. Members of phylum *Firmicutes* were lower in active CD than in controls and non-active CD groups. Members of phylum *Actinobacteria* were higher in active CD vs. non-active CD patients. *Klebsiella oxytoca* isolates were more prevalent in active CD than in controls. *Staphylococcus epidermidis* and *Staphylococcus pasteuri* were more prevalent in active CD group than in controls and inactive CD group. *Streptococcu anginosus* and *Streptococcus mutans* were more abundant in controls than in active and non-active CD. *Streptococcus mitis* group was higher in non-active CD than in active CD. *Actinomyces odontolyticus* was higher in active CD compared to inactive CD.
11	Di Biase et al. (30)	Overall, there was a dominance of *Enterobacteriaceae* (87.4%) in duodenal samples of CD patients. Sub-dominance of *Bacteroidetes* (4.8%) and *Streptococcus* (3%) was observed in several samples. The evaluation of the composition of the *Enterobacteriaceae* cluster was not possible due to the methodology used in the study. However, it was found that 50% of the samples had *Enterobacteriaceae* belonging to the genus *Proteus*.

**Table 4:** Main findings from the studies analyzing the fecal microbiota.

**#**	**References**	**Microbiome composition results**
1	Collado et al. (13)	*Bacteroides, Clostridium*, and *Staphylococcus* spp. were the dominant bacterial groups in CD patients, and their levels were higher than in controls. Dominant bacterial groups in controls were *Bifidobacterium* and *Enterobacteriaceae*. *Bacteroides-Prevotella, Clostridium histolyticum*, and *Clostridium coccoides* groups sulfate-reducing bacteria (SRB) and *Atopobium* group were significantly higher in CD patients than in controls.
2	Sanz et al. (15)	The fecal microbiota was more diverse in CD patients than in controls. In celiac children one to six different *Lactobacillus* groups were present; species other than *Lactobacillus* species were dominant in comparison with controls. The *Lactobacillus casei* group was more prevalent in control group than in celiac patients. *Bifidobacterium* species were significantly higher and diverse in controls than in CD patients. Controls combined both infant- and adult-type *Bifidobacterium* species, while CD patients mainly showed infant-type species (*Bifidobacterium bifidum* and *Bifidobacterium infantis*). *Bifidobacterium longum, Bifidobacterium pseudocatenulatum*, and *Bifidobacterium dentium* were higher in controls, while the *Bifidobacterium bifidum* was prevalent in CD group. *Bifidobacterium dentium* and *Bifidobacterium adolescentis* were not detected in any CD samples.
3	Collado et al. (16)	Fecal samples showed higher numbers of bifidobacteria than duodenal samples for every analyzed group of bacteria. *Bifidobacterium adolescentis* was detected more frequently in non-active CD than in active CD and controls. *Bifidobacterium dentium* was significantly more prevalent in non-active CD than in controls. The most predominant *Bifidobacterium* species in fecal samples were *Bifidobacterium longum, Bifidobacterium catenulatum*, and *Bifidobacterium bifidum*. Total *Bifidobacterium* levels were significantly higher in controls than in active and non-active CD patients.
4	Collado et al. (17)	Total bacterial counts were significantly lower in control children than in untreated and treated CD patients. *Staphylococcus* spp. were less prevalent in controls than in untreated and treated CD patients. *E. coli* was significantly higher in untreated and treated CD patients than in controls. *Bacteroides* and *Clostridium leptum* groups were significantly higher in both untreated and treated CD patients than in controls. *Bifidobacterium* spp. counts were significantly higher in controls than in untreated and treated CD patients. *Lactobacillus* spp. counts were significantly different only between treated CD patients and controls.
5	Di Cagno et al. (18)	*Bacteroides* and *Clostridium* were higher in treated CD and untreated CD than in controls. The ratio of lactic acid bacteria and *Bifidobacterium* to *Bacteroides* and enterobacteria, was lower in treated CD. This ratio was even lower in untreated CD. *Lactobacillus brevis, Lactobacillus rossiae*, and *Lactobacillus pentosus* were detected only in treated CD patients and controls. *Lactobacillus fermentum, Lactobacillus delbrueckii* subsp. *bulgaricus*, and *Lactobacillus gasseri* were observed only in several fecal samples of healthy controls. Compared to controls the composition of *Bifidobacterium* species varied for treated CD, while in untreated CD patients there was more variance of these species.
6	De Palma et al. (20)	Gram-positive bacterial population was more common in active CD patients compared to GFD celiac group. *Bifidobacterium* population was lower in fecal samples of untreated CD patients than in controls. Ig-A coated *Bacteroides-Prevotella* were higher in controls than in treated and untreated CD patients. *Clostridium histolyticum, Clostridium lituseburense*, and *Faecalibacterium prausnitzii* groups were lower in untreated CD than in controls. *E. coli, Staphylococcus, Lactobacillus-Enterococcus*, and sulfate-reducing bacteria were similar in all three groups studied.
7	Di Cagno et al. (22)	*Bacteroides, Porphyromonas*, and *Prevotella*, staphylococci/micrococci and *Enterobacteria* were more prevalent in fecal samples of treated CD patients. *Salmonella, Shigella* and *Klebsiella*, and *Clostridium* were not significantly different between the groups. Overall, the total anaerobes were the most prevalent in healthy controls. *Enterococcus* spp. was the largest group among lactic acid bacteria in both patients' groups. *Enterococcus faecium* was identified nearly in all fecal samples of both groups; *Enterococcus avium, Enterococcus faecalis, Enterococcus durans*, and *Enterococcus* spp. were also found. *Streptococcus macedonicus, Streptococcus pasteurianus, Pediococcus acidilactici*, and *Pediococcus pentosaceus* were identified in treated CD patients only. The most abundant species of lactobacilli in both groups of children were: *Lactobacillus plantarum, Lactobacillus casei*, and *Lactobacillus rhamnosus*. *Lactobacillus salivarius, Lactobacillus coryneformis, Lactobacillus delbrueckii* subsp. *bulgaricus, Lactobacillus fermentum*, and *Lactobacillus paracasei* were isolated in treated CD patients only. *Lactobacillus brevis, Lactobacillus pentosus*, and *Lactobacillus mucosae* were only found in controls. *Enterococcus* was largest genus isolated among lactic acid bacteria for both patients' groups.
8	Sánchez et al. (25)	*Staphylococcus epidermidis* was lower in the control group than in active and non-active CD groups. *Staphylococcus haemolyticus* was higher in active CD group than in controls. *Staphylococcus aureus* was lower in active CD children compared to non-active CD and controls. *Staphylococcus* spp. was more diverse in active CD patients than in all the other patients.
9	Pisarello et al. (28)	Lactobacilli were less abundant in the CD patients on GFD than in control children. The enterobacteria population had a trend to increase in CD group compared to healthy controls. Still, there was no significant differences in total counts of the aerobic and anaerobic among two studied groups.
10	Quagliariello et al. (29)	*Lactobacillus* spp. was more abundant in controls than in CD patients. *Bacteroides fragilis* was higher in CD group than in controls. No significant differences were detected in levels of *Bacteroides*. After the treatment with probiotics, the enterobacteria were higher in the controls compared to CD group. Later, after 3 months of probiotic treatment, the levels of enterobacteria declined in the probiotic group.
11	Di Biase et al. (30)	A statistically significant lower relative abundance was detected for *Bacteroides/Prevotella* cluster in CD group (10.2%) compared to the control group (15.6%). A statistically significant lower abundance in the cluster *Akkermansia* was found in CD group (0.7%) compared to control patients (4.2%) and, as for the cluster *Staphylococcaceae*, in CD group (0.9%) compared to healthy controls (2%). The relative abundances of bacterial clusters of duodenal and fecal microbiota were not statistically significantly correlated.

## Discussion

In the landscape of immune-mediated non-communicable disorders, CD is the only disease for which the necessary HLA genetic background and environmental trigger are known. Briefly, the dietary intake of gluten triggers the development of CD in some of those individuals who are carriers of the specific HLA-DQ allelic variants coding DQ2 and DQ8 heterodimers (DQA1*0501-DQB1*02 and DQA1*0301-DQB1*0302, respectively) (4, 5). Overall, 30–40% of the general population in Europe and North America carry this HLA-DQ genotype, but only 3% of them actually develop CD during the life (despite the dietary exposure to gluten foods), which thus corresponds to around 1% prevalence in the general population (31, 32). Therefore, these HLA-DQ genes and the gluten dietary intake, even if necessary factors, are not sufficient to develop CD; other concomitant (and currently unknown or not well-defined) environmental agents, non-HLA genetic aspects, and maybe epigenetic mechanisms, are supposed to play a critical role in determining which individuals will become celiac within a much larger HLA-predisposed and gluten-exposed population. In this regard, the gut microbiota has been considered among the factors potentially affecting or modulating the risk of developing CD, through its interplay with the intestinal epithelium and/or the host immune system (33, 34).

In this systematic review, we aimed at summarizing and describing the main characteristics of the gut microbiota in CD children, compared to non-celiac controls. In order to achieve this purpose, we analyzed the pediatric studies investigating both fecal samples and duodenal specimens, which present substantial differences, of course. Indeed, as mentioned, the gut microbiota greatly varies along the GI tract, due to the different environmental conditions, in terms of pH, oxygen tension, substrates availability, host secretion, and intestinal motility (35). In the duodenum (which is characterized by a relatively acidic pH, high level of oxygen, and rapid transit time), facultative anaerobic and rapidly growing bacteria able to adhere to the epithelium in the mucus layer, are more likely to survive. On the contrary, the fecal samples are more representative of the colonic environment, which presents more favorable conditions for bacterial survival and, thus, is characterized by a more abundant and diverse microbiota, including mainly anaerobes (36, 37).

Eleven pediatric studies provided information on the duodenal microbiota of untreated CD children and age-matched controls. Among those, eight included only pediatric patients (14, 16, 17, 19, 21, 22, 27, 30), whereas three investigated both children and adults (23, 24, 26).

Nadal et al. (14) mainly found that *Bacteroides* spp., *Prevotella* spp., and *Escherichia coli* populations were significantly more abundant in CD patients with active disease than in controls. Recently, Di Biase et al. (30) showed a “total dominance” of *Enterobacteriaceae* in the duodenal flora of active CD children; to follow, *Bacteroidetes* spp. and *Streptococcus* spp. turned out to be the second most represented category. However, no control group was included for this analysis. Schippa et al. (21) described a significantly higher microbiota biodiversity in CD children, compared to controls; in terms of bacterial composition, these authors highlighted a significant difference in the prevalence of *Bacteroides vulgatus* (85 vs. 20%) and *E. coli* (95 vs. 20%) between these two groups. In terms of phyla, as described by Sánchez et al. (27), Firmicutes and Proteobacteria were respectively more and less abundant in children with CD, compared to their controls; importantly, these results were also confirmed with respect to CD patients on gluten-free diet (non-active CD group).

Actually, two of the aforementioned studies also included non-active CD children [Nadal et al. (14) and Schippa et al. (21)]: both found no significant differences between these groups of children and their controls, in terms of bacterial proportions and biodiversity, respectively. Overall, all these three studies on pediatric CD indicated that the bacterial deviations could be normalized after the appropriate gluten-free diet. Conversely, Collado et al. (17) described significantly higher number of Bacteroides and *Clostridium leptum* in both treated and untreated (active) CD children compared to controls; however, a statistically significant difference between active CD children from one side, and both treated CD and control children, on the other side, was reported for *Staphylococcus* spp. and *E. coli*.

Three additional studies included adult CD patients, in addition to children. Actually, Kalliomäki et al. (24) compared CD children to healthy children and non-active CD adults: they found no difference in bacterial counts among these three groups. Again, in the study by Nistal et al., there was no significant difference between active CD and healthy controls, considering the 36 different genera of known bacteria detected by their analysis. Importantly, this study provided a direct comparison between CD children and adults: even though both types of patients were colonized by bacteria mainly belonging to the Firmicutes, Proteobacteria, and Bacteroidetes phyla, the bacterial abundance and diversity were significantly lower in CD children than in CD adults (23). Cheng et al. also reported that the overall composition and diversity of gut microbiota was comparable between CD children and healthy controls; however, the specific profiles of eight bacterial groups were significantly different: in detail, *Prevotella* spp. (and, in particular, *Prevotella melaninogenica*), *Haemophilus* spp., and *Serratia* spp. (and, in particular, *S. marcescens*) were more abundant in CD children, whereas *Prevotella oralis, Ruminococcus bromii, Papillibacter cinnamivorans, Proteus* spp., and *Clostridium stercorarium* were less abundant (26).

Importantly, several studies focused the attention on some specific bacteria and, in detail, *Bifidobacterium* spp. and *Lactobacillus* spp. As for the former group, in both studies by Collado et al. (16), a significantly lower number was reported in CD (and, in particular, untreated) children than in controls; on the contrary, Di Cagno et al. (18) found no significant representation of Bifidobacteria in their CD and control groups, overall. No significant differences were reported in the prevalence of *Bifidobacterium* spp. between CD patients and controls, and between active and inactive CD, in the study by Schippa et al. (21). As regards *Lactobacillus* spp., Di Cagno et al. (18) described a relatively homogeneous population in all children, whereas Collado et al. (16) reported that these bacteria were significantly less abundant in controls and treated CD children than in the active CD group.

Therefore, the available data on duodenal microbiota in CD children are not all consistent among themselves. The main limitation of the aforementioned studies is the small sample size, since all these studies included around or no more than 30 children, probably due to technical and ethical issues concerning the invasive procedures required to analyze the duodenal microbiota. This aspect may have also affected the selection and inclusion of an appropriate control group, of course: actually, whereas in the studies by Collado et al. (13) and Nadal et al. (14) these control children are not better defined than as “without gluten intolerance” and “without known food intolerance,” only Schippa et al. (21) disclosed the reason why they underwent upper GI endoscopy, namely, “functional dyspepsia.” Therefore, the unclear description and, anyway, the impossibility to compare these findings with completely healthy children represent additional obstacles to the appropriate interpretation of the microbiota findings obtained from the duodenal mucosa.

These specific research limitations can be lessened by studying the microbiota in fecal samples, whose collection does not imply any invasive procedure, of course. Importantly, this aspect makes this analysis much more attractive as potential non-invasive diagnostic and/or prognostic biomarkers for pediatric CD.

Eleven pediatric studies provided information on the fecal microbiota of untreated CD children and age-matched controls. Among those, all investigated only pediatric patients (13, 16–18, 20, 22, 25, 28–30), except one, which included both children and adults (15).

To start with, Collado et al. (13) showed significantly lower total bacterial counts in stools from the control group compared to treated or untreated CD children, but this specific information was not provided by other studies. In terms of microbiota general diversity, Sanz et al. (15) found it significantly higher in CD children than in controls, but Di Cagno et al. (18) could not confirm this finding.

Some studies mainly analyzed the relative composition of the fecal microbiota. In general, De Palma et al. (20) investigated both active and treated CD children, in addition to healthy controls: they reported a significantly decreased gram+/gram– ratio in both groups of CD children, compared to controls; interestingly, treated CD children showed intermediate values between active CD children and healthy controls, but the difference was statistically significant only with the latter group, suggesting that this specific aspect was not completely restored by the gluten-free diet. In terms of phyla, these authors reported that the Bacteroides/Prevotella cluster was significantly more expressed in untreated CD patients than in healthy controls; this finding was confirmed by Collado et al. (16, 17) and Quagliariello et al. (29).

However, most researchers investigated some specific bacteria of the fecal microbiota, mainly *Bifidobacterium* spp. and *Lactobacillus* spp., which differed in terms of relative species abundance between CD patients and healthy controls (15, 22, 28). Sánchez et al. (25) focused on *Staphylococcus* spp. and observed some peculiarities in species diversity and abundance in CD children; however, the increased presence of *S. epidermidis* isolates carrying genes conferring resistance to methicillin suggested that these changes may be induced by a greater exposure to antibiotics rather than CD itself. However, Di Biase et al. (30) reported a lower abundance of *Staphylococcus* spp. in CD children, as well as a significant reduction of the Bacteroides/Prevotella cluster, which is in contrast with some of the aforementioned studies.

Therefore, even though the available fecal microbiota studies in CD children are more than those investigating the duodenal microbiota, their clinical aims and settings were quite heterogeneous: indeed, they provided a qualitative and quantitative information that is not immediately comparable, and the results are often conflicting because of that, probably.

Importantly, 3 of these 11 articles on fecal microbiota also provided data related to duodenal biopsies from the same cohorts of patients (16, 17, 30). In detail, Collado et al. discussed the relation between fecal and duodenal microbiota: they found significant correspondences for *Bifidobacterium* spp. among all three groups (active CD, treated CD, and controls), whereas other bacterial groups did not correlate in all groups. As regards specifically untreated CD children, *Bacteroides* spp., *Staphylococcus* spp., *Lactobacillus* spp., *C. leptum* group, *Clostridium coccoides* group, *E. coli*, and *Akkermansia muciniphila* showed a significant correlation between these two types of samples (16, 17). Unfortunately, Di Biase et al. (30) did not analyze such a correlation; however, about this study, it may be worth to emphasize the fact that *A. muciniphila* was found to be significantly less abundant in CD children's stool, along with *Bacteroides* spp. and the *Staphylococcus* group, as previously mentioned. Although Collado et al. (13) described a significant duodenal–fecal relation for that microorganism, they did not actually find statistically significant differences in its content among their study groups. In an additional study, they focused on *Bifidobacterium* spp. and, once again, they compared duodenal biopsies and fecal samples in the same cohort of patients. The fecal microbiota resulted to include significantly higher numbers of bifidobacteria than the duodenal samples. Interestingly, they found a statistically significant correlation between these sample types in all groups as regards the total number of bifidobacteria and, in terms of species, only *Bifidobacterium longum* significantly correlated in both samples and all groups. *B. longum* was the most frequent and abundant species in this study and, importantly, was significantly different among all study groups, both in stools and duodenum, in addition to be the only one to significantly correlate between both types of samples, as previously explained (16).

Indeed, *B. longum* was the object of some preliminary investigations as potential therapeutic resource and prognostic biomarker. In a double-blind, randomized, and placebo-controlled trial, Olivares et al. (38) showed that the administration of the CECT 7347 strain of *B. longum* was associated with a significant reduction of the Bacteroides fragilis group in the microbiota of CD patients, in addition to some clinical benefit in terms of anthropometric parameters. Some recent analyses derived from a large perspective cohort study (PROFICEL), comparing the fecal microbiota in children at HLA-DQ genetic risk of CD before the appearance of the disease (exactly, at 4 and 6 months of life), highlighted some microbiota differences between those children who eventually developed CD and those who did not. Interestingly, high-risk genotypes for CD (referring to HLA-DQ2 and HLA-DQ8 heterodimers) were associated to a lower amount of *Bifidobacterium* spp. and, specifically, *B. longum* (39–41). More in general, several microbial species and related metabolites (with inflammatory and immunological properties) have been recently suggested as potentially specific to CD, through multi-omics analysis (42).

Even though the interplay between HLA-DQ (and, in detail, HLA-DQB1*02, which is the most frequent allelic variant in CD children) and intestinal microbiota must be precisely elucidated yet, these preliminary observations might provide the background to plan further studies to assess the risk of developing CD in gluten-exposed population and, potentially, to consider additional non-invasive diagnostic tools and prognostic markers for CD (37, 43, 44).

## Conclusion

Due to the heterogeneity of the experimental procedures and design of these studies, it is not possible to evidence any specific and absolute celiac signature in the fecal and/or duodenal microbiota of CD children. However, some specific components of the fecal microbiota (e.g., *Bifidobacterium* spp.) may deserve additional research efforts to understand the potential application as both probiotic therapy and/or diagnostic/prognostic biomarker.

## Data Availability Statement

The original contributions presented in the study are included in the article/supplementary material, further inquiries can be directed to the corresponding author/s.

## Author Contributions

DP and DA conceived the study and wrote the review. DA, DP, and KD performed the literature search. All authors contributed to the article and approved the submitted version.

## Conflict of Interest

The authors declare that the research was conducted in the absence of any commercial or financial relationships that could be construed as a potential conflict of interest.

## References

[1] Nardone G Compare D Rocco A. A microbiota-centric view of diseases of the upper gastrointestinal tract. Lancet Gastroenterol Hepatol. (2017) 2:298–312. 10.1016/S2468-1253(16)30108-X 28404159

[2] Dieterich W Schink M Zopf Y. Microbiota in the gastrointestinal tract. Med Sci. (2018) 6:116. 10.3390/medsci6040116 PMC6313343 30558253

[3] Lindfors K Ciacci C Kurppa K Lundin KEA Makharia GK Mearin ML. Coeliac disease. Nat Rev Dis Prim. (2019) 5:3. 10.1038/s41572-018-0054-z 30631077

[4] Lebwohl B Sanders DS Green PHR. Coeliac disease. Lancet. (2018) 391:70–81. 10.1016/S0140-6736(17)31796-8 28760445

[5] Poddighe D Rebuffi C de Silvestri A Capittini C. Carrier frequency of HLA-DQB1*02 allele in patients affected with celiac disease: a systematic review assessing the potential rationale of a targeted allelic genotyping as a first-line screening. World J Gastroenterol. (2020) 26:1365–81. 10.3748/wjg.v26.i12.1365 32256023 PMC7109277

[6] Rinninella E Mele MC Merendino N Cintoni M Anselmi G Caporossi A. The role of diet, micronutrients and the gut microbiota in age-related macular degeneration: new perspectives from the gut–retina axis. Nutrients. (2018) 10:1677. 10.3390/nu10111677 PMC6267253 30400586

[7] Arumugam M Raes J Pelletier E Le Paslier D Yamada T Mende DR. Enterotypes of the human gut microbiome. Nature. (2011) 473:174–80. 10.1038/nature09944 21508958 PMC3728647

[8] Thursby E Juge N. Introduction to the human gut microbiota. Biochem J. (2017) 474:1823–36. 10.1042/BCJ20160510 28512250 PMC5433529

[9] Vuik FER Dicksved J Lam SY Fuhler GM van der Laan L van de Winkel A. Composition of the mucosa-associated microbiota along the entire gastrointestinal tract of human individuals. United Eur Gastroenterol J. (2019) 7:897–907. 10.1177/2050640619852255 31428414 PMC6683645

[10] Cho I Blaser MJ. The human microbiome: at the interface of health and disease. Nat Rev Genet. (2012) 13:260–70. 10.1038/nrg3182 22411464 PMC3418802

[11] Sommer F Bäckhed F. The gut microbiota-masters of host development and physiology. Nat Rev Microbiol. (2013) 11:227–38. 10.1038/nrmicro2974 23435359

[12] Moher D Liberati A Tetzlaff J Altman DG. Preferred reporting items for systematic reviews and meta-analyses: the PRISMA statement. J Clin Epidemiol. (2009) 62:1006–12. 10.1016/j.jclinepi.2009.06.005 19631508

[13] Collado MC Calabuig M Sanz Y. Differences between the fecal microbiota of coeliac infants and healthy controls. Curr Issues Intest Microbiol. (2007) 8:9–14. 17489434

[14] Nadal I Donant E Ribes-Koninckx C Calabuig M Sanz Y. Imbalance in the composition of the duodenal microbiota of children with coeliac disease. J Med Microbiol. (2007) 56:1669–74. 10.1099/jmm.0.47410-0 18033837

[15] Sanz Y Sánchez E Marzotto M Calabuig M Torriani S Dellaglio F. Differences in faecal bacterial communities in coeliac and healthy children as detected by PCR and denaturing gradient gel electrophoresis. FEMS Immunol Med Microbiol. (2007) 51:562–8. 10.1111/j.1574-695X.2007.00337.x 17919298

[16] Collado MC Donat E Ribes-Koninckx C Calabuig M Sanz Y. Imbalances in faecal and duodenal Bifidobacterium species composition in active and non-active coeliac disease. BMC Microbiol. (2008) 8:232. 10.1186/1471-2180-8-232 19102766 PMC2635381

[17] Collado MC Donat E Ribes-Koninckx C Calabuig M Sanz Y. Specific duodenal and faecal bacterial groups associated with paediatric coeliac disease. J Clin Pathol. (2009) 62:264–9. 10.1136/jcp.2008.061366 18996905

[18] Di Cagno R Rizzello CG Gagliardi F Ricciuti P Ndagijimana M Francavilla R. Different fecal microbiotas and volatile organic compounds in treated and untreated children with celiac disease. Appl Environ Microbiol. (2009) 75:3963–71. 10.1128/AEM.02793-08 19376912 PMC2698361

[19] Sánchez E Donat E Ribes-Koninckx C Calabuig M Sanz Y. Intestinal bacteroides species associated with coeliac disease. J Clin Pathol. (2010) 63:1105–11. 10.1136/jcp.2010.076950 20972239

[20] De Palma G Nadal I Medina M Donat E Ribes-Koninckx C Calabuig M. Intestinal dysbiosis and reduced immunoglobulin-coated bacteria associated with coeliac disease in children. BMC Microbiol. (2010) 10:63. 10.1186/1471-2180-10-63 20181275 PMC2843610

[21] Schippa S Iebba V Barbato M Di Nardo G Totino V Checchi MP. A distinctive “microbial signature” in celiac pediatric patients. BMC Microbiol. (2010) 10:175. 10.1186/1471-2180-10-175 20565734 PMC2906462

[22] Di Cagno R De Angelis M De Pasquale I Ndagijimana M Vernocchi P Ricciuti P. Duodenal and faecal microbiota of celiac children: molecular, phenotype and metabolome characterization. BMC Microbiol. (2011) 11:219. 10.1186/1471-2180-11-219 21970810 PMC3206437

[23] Nistal E Caminero A Herrán AR Arias L Vivas S de Morales JM. Differences of small intestinal bacteria populations in adults and children with/without celiac disease: effect of age, gluten diet, and disease. Inflamm Bowel Dis. (2012) 18:649–56. 10.1002/ibd.21830 21826768

[24] Kalliomäki M Satokari R Lähteenoja H Vähämiko S Grönlund J Routi T. Expression of microbiota, toll-like receptors, and their regulators in the small intestinal mucosa in celiac disease. J Pediatr Gastroenterol Nutr. (2012) 54:727–32. 10.1097/MPG.0b013e318241cfa8 22134550

[25] Sánchez E Ribes-Koninck C Calabuig M Sanz Y. Intestinal *staphylococcus* spp. and virulent features associated with coeliac disease. J Clin Pathol. (2012) 65:830–4. 10.1136/jclinpath-2012-200759 22718843

[26] Cheng J Kalliomäki M Heilig HGHJ Palva A Lähteenoja H de Vos WM. Duodenal microbiota composition and mucosal homeostasis in pediatric celiac disease. BMC Gastroenterol. (2013) 13:113. 10.1186/1471-230X-13-113 23844808 PMC3716955

[27] Sánchez E Donat E Ribes-Koninckx C Fernández-Murga ML Sanz Y. Duodenal-mucosal bacteria associated with celiac disease in children. Appl Environ Microbiol. (2013) 79:5472–9. 10.1128/AEM.00869-13 23835180 PMC3754165

[28] Pisarello MLJ Vintiñi EO González SN Pagani F Medina MS. Decrease in lactobacilli in the intestinal microbiota of celiac children with a gluten-free diet, and selection of potentially probiotic strains. Can J Microbiol. (2014) 61:32–7. 10.1139/cjm-2014-0472 25438612

[29] Quagliariello A Aloisio I Bozzi cionci N Luiselli D D'Auria G Martinez-Priego L. Effect of bifidobacterium breve on the intestinal microbiota of coeliac children on a gluten free diet: A pilot study. Nutrients. (2016) 8:660. 10.3390/nu8100660 27782071 PMC5084046

[30] Di Biase AR Marasco G Ravaioli F Dajti E Colecchia L Righi B. Gut microbiota signatures and clinical manifestations in celiac disease children at onset: a pilot study. J Gastroenterol Hepatol. (2020) 36:446–54. 10.1111/jgh.15183 32666516

[31] Ludvigsson JF Murray JA. Epidemiology of celiac disease. Gastroenterol Clin North Am. (2019) 48:1–8. 10.1016/j.gtc.2018.09.004 30711202

[32] Poddighe D Rakhimzhanova M Marchenko Y Catassi C. Pediatric celiac disease in central and east Asia: Current knowledge and prevalence. Medicina (Kaunas). (2019) 55:11. 10.3390/medicina55010011 30642036 PMC6359221

[33] Valitutti F Cucchiara S Fasano A. Celiac disease and the microbiome. Nutrients. (2019) 11:2403. 10.3390/nu11102403 PMC6835875 31597349

[34] Krishnareddy S. The microbiome in celiac disease. Gastroenterol Clin North Am. (2019) 48:115–26. 10.1016/j.gtc.2018.09.008 30711204

[35] Flint HJ Scott KP Louis P Duncan SH. The role of the gut microbiota in nutrition and health. Nat Rev Gastroenterol Hepatol. (2012) 9:577–89. 10.1038/nrgastro.2012.156 22945443

[36] Donaldson GP Lee SM Mazmanian SK. Gut biogeography of the bacterial microbiota. Nat Rev Microbiol. (2015) 14:20–32. 10.1038/nrmicro3552 26499895 PMC4837114

[37] Gu S Chen D Zhang JN Lv X Wang K Duan LP. Bacterial community mapping of the mouse gastrointestinal tract. PLoS ONE. (2013) 8:e74957. 10.1371/journal.pone.0074957 24116019 PMC3792069

[38] Olivares M Castillejo G Varea V Sanz Y. Double-blind, randomised, placebo-controlled intervention trial to evaluate the effects of Bifidobacterium longum CECT 7347 in children with newly diagnosed coeliac disease. Br J Nutr. (2014) 112:30–40. 10.1017/S0007114514000609 24774670

[39] Olivares M Walker AW Capilla A Benítez-Páez A Palau F Parkhill J. Gut microbiota trajectory in early life may predict development of celiac disease. Microbiome. (2018) 6:36. 10.1186/s40168-018-0415-6 29458413 PMC5819212

[40] Rintala A Riikonen I Toivonen A Pietilä S Munukka E Pursiheimo JP. Early fecal microbiota composition in children who later develop celiac disease and associated autoimmunity. Scand J Gastroenterol. (2018) 53:403–9. 10.1080/00365521.2018.1444788 29504486

[41] De Palma G Capilla A Nova E Castillejo G Varea V Pozo T. Influence of milk-feeding type and genetic risk of developing coeliac disease on intestinal microbiota of infants: the PROFICEL study. PLoS ONE. (2012) 7:e30791. 10.1371/journal.pone.0030791 22319588 PMC3272021

[42] Leonard MM Karathia H Pujolassos M Troisi J Valitutti F Subramanian P. Multi-omics analysis reveals the influence of genetic and environmental risk factors on developing gut microbiota in infants at risk of celiac disease. Microbiome. (2020) 8:130. 10.1186/s40168-020-00906-w 32917289 PMC7488762

[43] Poddighe D Turganbekova A Baymukasheva D Saduakas Z Zhanzakova Z Abdrakhmanova S. Genetic predisposition to celiac disease in Kazakhstan: potential impact on the clinical practice in Central Asia. PLoS ONE. (2020) 15:e0226546. 10.1371/journal.pone.0226546 31895924 PMC6939901

[44] Capittini C De Silvestri A Rebuffi C Tinelli C Poddighe D. Relevance of HLA-DQB1*02 allele in the genetic predisposition of children with celiac disease: additional cues from a meta-analysis. Medicina. (2019) 55:190. 10.3390/medicina55050190 31121940 PMC6571594

